# Congenital granular cell tumor: Report of a case with literature review and differential diagnosis

**DOI:** 10.1002/ccr3.5580

**Published:** 2022-03-13

**Authors:** Arpita Singh, Snehashish Ghosh, Anjani Kumar Yadav, Anuja Panthee

**Affiliations:** ^1^ Department of Oral and Maxillofacial Surgery National Medical College and Teaching Hospital Birgunj Nepal; ^2^ Department of Oral Pathology College of Medical Sciences Bharatpur Nepal; ^3^ Department of Oral and Maxillofacial Surgery B.P. Koirala Institute of Health Sciences Dharan Nepal; ^4^ Department of Oral and Maxillofacial Surgery KIST Medical College and Teaching Hospital Kathmandu Nepal

**Keywords:** fibrous, mass, Neumann's tumor, size

## Abstract

Congenital granular cell tumor (CGCT) is a rare benign lesion and presents as a fibrous mass arising from the alveolus in the newborn. The prenatal screening of lesions can help in parent counseling, determining the complications, as larger size lesions may interfere with normal delivery and require a cesarean section.

## Graphical abstract

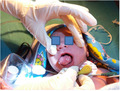

## 1 INTRODUCTION

Congenital granular cell tumor is a rare benign soft tissue tumor that originates from the gingiva of the maxillary or mandibular alveolar ridge in the newborn. It was first described by Ernst Christian Neumann in 1871 as “Congenital Epulis.”^1^ The other names for CGCT are Neumann's tumor, Congenital Epulis, Congenital Myoblastoma, and Gingival Granular Cell Tumor.^2^ The term “epulis” simply means swelling on the gingival thus it was suggested to be discontinued and congenital granular cell tumor to be used in the literature. The incidence of occurrence of the tumor is very low (0.0006%).^2^

The congenital granular cell tumor is seen twice as commonly involving maxillary alveolar ridge to mandibular ridge with the incisor canine region being the most prevalent site.^1^, ^2^ The lesion is predominant in females to males. The histogenesis of this tumor is not very clear, several studies have been done but none is conclusive thus various authors have proposed different sources of origin of this tumor.^2^, ^3^ Prenatal ultrasound imaging could give some clue about its presence, which can alert the gynecologists about the potential complications that can arise during delivery.^3^ The probable source of origin suggested is from undifferentiated mesenchymal cells,^4^ odontogenic epithelial cells, pericytic, fibroblastic, histiocytes, nerve‐related, smooth muscle, and primitive mesenchymal cells.^4^, ^5^, ^6^

We here report a case of congenital granular cell tumor (CGCT) in the incisor region of the lower alveolar ridge. This case report is unique, owing to the size of the tumor, which is relatively rare, along with it we tried to highlight the differential diagnosis of this particular entity in a separate table which will guide the clinicians to arrive at a correct diagnosis when they encounter with such a lesion.

## 2 CASE REPORT

**FIGURE 1:**
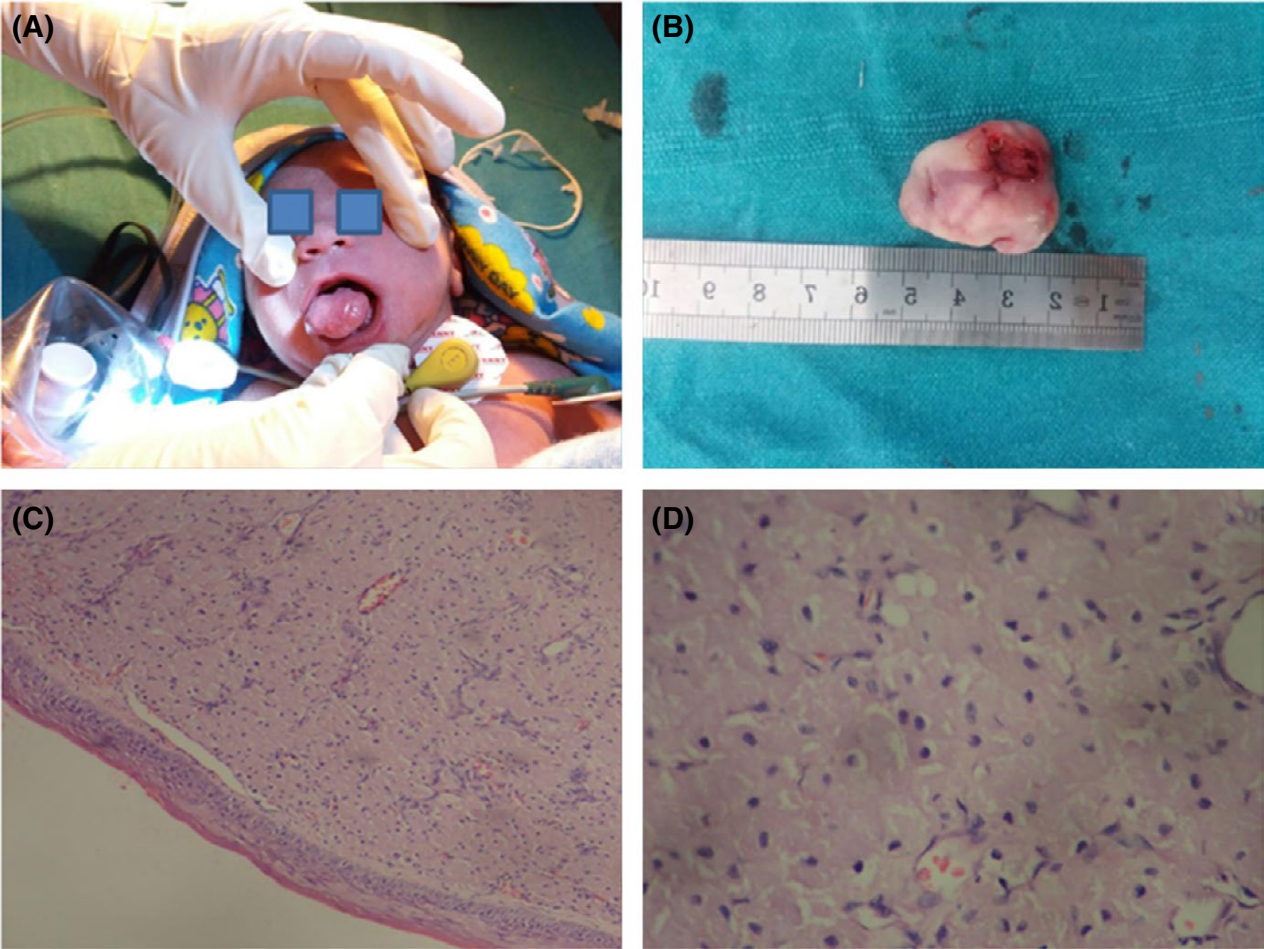
(A) Examination of the case of congenital granular cell tumor (CGCT), (B) Excised specimen of CGCT in one‐day‐old neonate, (C) Granular cells in a fibrovascular stroma, lined by thin, atrophic epithelium (H&E‐40× magnification), (D) Large, round granular cells with basophilic nuclei and abundant eosinophilic cytoplasm (H&E‐100× magnification)

A newborn female patient was referred to the faculty of dentistry, National Medical College and teaching hospital, Birgunj, Nepal. The child had a normal delivery in the maternity department of the same institution and was referred to us 5 h post‐delivery for evaluation and management of mass in the mouth. On clinical examination, the lesion presented with a pedunculated, smooth‐surfaced mass, which was pink in color, size about 2.5 cm in diameter on the anterior part of the mandibular alveolus (Figure 1A). The tumor mass was relatively large, which was interfering with the mouth closure, causing difficulty with breastfeeding, which was distressing both for the patient and the parent. The provisional diagnosis of the granular cell tumor was given for the lesion with the differential diagnosis of embryonal rhabdomyosarcoma and melanotic neuroectodermal tumor of infancy. As the lesion was not pulsatile, hemangioma was excluded from the differential diagnosis.

### 2.1 Pre‐operative evaluation

Pediatric consultation was done for the child and routine blood investigations like complete blood count, bleeding time, clotting time, the international normalized ratio was done. The reports were found to be within the normal limits. A single dose of 1‐mg vitamin K injection was administered intravenously to prevent vitamin K deficiency bleeding.

### 2.2 Surgical intervention

The surgery was performed under intravenous anesthesia Local anesthetic (2% lignocaine) was infiltrated at the base of the lesion to decrease intra‐operative bleeding and post‐operative pain. The lesion was excised with electrocautery. The excised mass (2.3 × 1.8 × 1.3) cm (Figure 1B) was fixed in 10% neutral\‐buffered formalin and sent for histopathological examination.

### 2.3 Histopathologic examination

The multiple sections of hematoxylin and eosin‐stained tissues revealed stratified squamous epithelium lining the lesion showing atrophy of rete ridges and the underlying stroma bears unencapsulated tumor composed of sheets of large, polygonal, round, and oval cells with indistinct cell border, granular, eosinophilic cytoplasm, and round to oval lightly basophilic nuclei (Figure 1C,D). The microscopic findings were consistent with features of CGCT.

### 2.4 Post‐operative evaluation

The child was observed postoperatively for 3 h. Feeding was done with the spoon, which she tolerated well. A pediatric review was done, and then, the child was discharged. As the patient resided far from the hospital, the patient was never brought to the hospital for the follow‐up. However, the child's father reported to the department 15 days post‐surgery, stating that the child is doing well. As there is no concrete follow‐up data, the authors consider it as a limitation of this case report.

## 3 DISCUSSION

The clinical course of CGCT is not clear but still, it is considered as a benign lesion and does not grow postpartum. It was suggested that trauma due to finger sucking in utero could be a possible cause for the CGCT but it had little evidence so the concept was discarded. The most recent concept about CGCT is it is either a reactive lesion. Mostly it presents as an isolated lesion but there are reports of it presenting simultaneously with other entities, chiefly neurofibromatosis, transverse facial cleft, and blinder syndrome.^4^, ^6^ Rarely it is associated with congenital missing of the tooth germ in the region of its occurrence. Therefore, as it is a congenital entity, it is associated with prenatal and post‐natal complications.^4^, ^6^

The clinical diagnosis of CGCT can be made by the presence of a fibrous mass in the alveolar mucosa of the mandible or maxilla at the time of birth.^7^ Clinically the tumor is smooth or lobular surfaced, firm to rubbery in consistency.^6^, ^8^ Typically CGCT occurs as a single tumor but case reports with multiple lesions have also been reported involving either one or both jaws. Other abnormalities such as nasal bridges, neurofibroma, polydactyly, Binder syndrome, congenital goiter can be seen with multiple CGCT.^4^, ^9^, ^10^ The size of the lesion may also determine the intervention planning as a large lesion may interfere with normal delivery and may require a cesarean section.^3^, ^9^ The tumor has no familial tendency, surgical excision remains the treatment of choice for CGCT; spontaneous regression has also been reported.^4^, ^9^ Recurrence or malignant transformation for CGCT has not yet been reported in the literature.^4^

Prenatal diagnosis of CGCT can be done by ultrasonography as early as 26 weeks of pregnancy.^9^ The commonest prenatal complication associated with CGCT is obstructed deglutition of amniotic fluid, whereas after birth it could be associated with hypoplasia of incisors, midface hypoplasia, feeding difficulties, and respiratory obstruction.^3^, ^9^ Hence, its diagnosis prenatally is important by ultrasonography, can help in parent counseling regarding the nature and management of disease post‐delivery.^4^, ^10^ Review of literature of the accessible 24 cases of CGCT reported in the last two decades, including the present case is mentioned in Table 1.

**TABLE 1:** Review of literature of the accessible 24 cases of congenital granular cell tumor (CGCT) reported in the last two decades, including the present case

Serial number	Author (Year)	Age/Sex	Presentation/Size	Region affected	Treatment	Follow‐up	Healing/Recurrence
1.	BL Koch et al. (1997)^16^	Newborn/Gender not specified	Pedunculated gingival mass/Maxillary‐(2.9 cm in greatest dimension).	Maxillary and mandibular alveolar ridge.	Excision.	Follow‐up time not mentioned.	Healing uneventful, recurrence data not mentioned.
2.	Reinshagen K et al. (2002)^17^						
	Case 1	Newborn/Female.	Soft tissue tumor/(1.8 × 1.3 × 0.8) cm.	Maxillary right alveolar ridge.	Electric cauterization.	Follow‐up time not mentioned.	Healing uneventful, no recurrence.
	Case 2	4‐week‐old/Female.	Pedunculated soft tissue tumor/(1.2 × 0.6 × 0.5) cm.	Maxillary left alveolar ridge.	Electric cauterization.	Follow‐up time is not mentioned.	Not known.
3.	Merrett SJ et al. (2003)^18^	Newborn/Female.	Pedunculated soft tissue mass/(1.5 × 1.4 × 1.4) cm.	Maxillary left alveolar ridge.	Excision.	2 weeks.	Healing uneventful, no recurrence.
4.	Kanotra S et al. (2005)^19^	5‐day‐old/Female.	Pedunculated mass with surface ulceration/(5 × 3 × 2.5) cm.	Mandibular alveolar ridge.	Excision.	2 years.	Healing uneventful, no recurrence.
5.	Silva GG (2007)^20^	3‐day‐old/Female.	Bilobed, pedunculated mass/(Diameter–2 cm).	Maxillary anterior alveolar region.	Electric cauterization.	Follow‐up time is not mentioned.	Healing uneventful, recurrence data not mentioned.
6.	Eghbalian F et al (2009)^21^	Newborn/Female.	Two soft tissue lesions/(4.5 × 3.3) cm and (1.5 × 1) cm.	Maxillary alveolar ridge.	Excision.	6 months.	Healing uneventful, no recurrence.
7.	M Al Ani et al. (2010)^22^	Newborn/Female.	Pedunculated soft tissue mass/(2 × 1) cm.	Maxillary anterior alveolar ridge.	Electric cauterization.	10 days	Healing uneventful, no recurrence.
8.	D Steckler Jr et al (2011)^23^	Newborn/Sex Not specified.	2 soft tissue mass/(4 × 3 × 2) cm, (1 × 1) cm.	Maxillary gingiva.	Excision.	6 months.	Healing uneventful/No recurrence.
9.	B Sigdel et al (2011)^24^	Newborn/Female.	Slightly lobulated angiomatous mass/(4 × 3 × 2) cm.	Maxillary alveolar ridge.	Excision.	Regular follow‐up (Exact duration is not specified).	Healing uneventful/No recurrence.
10.	Aparna HG et al. (2014)^25^	Newborn/Female.	Solitary, round soft pedunculated mass/(3.5 × 3.5 × 2) cm.	Maxillary alveolar ridge/	Excision.	Follow‐up time is not mentioned.	Healing uneventful, recurrence data not mentioned.
11.	Saki N et al. (2014)^26^	Newborn/Female.	Multiple soft tissue lesions/ Maxillary (2 × 1.5 × 1) cm, (1 × 0.8 × 0.5) cm, Mandibular (1 × 0.5 × 0.4) cm.	2 Maxillary and 1 mandibular alveolar ridge.	Excision.	Follow‐up time is not mentioned.	Healing uneventful, recurrence data not mentioned.
12.	Liang Y et al. (2014)^27^	4‐day‐old/Female.	Multiple, pedunculated soft tissue lesions/(Size of largest–3.5 × 3) cm.	2 on maxillary, 4 on mandibular alveolar ridge.	Excision.	2 months.	Healing uneventful, no recurrence.
13.	A Aresdahl et al (2015)^28^	Newborn/Female.	Large soft tissue mass/(2 × 2) cm.	Right maxillary alveolar process.	Excision.	6 months.	Healing uneventful/No recurrence.
14.	RM Kumar et al. (2015)^29^	3‐day‐old/Female.	Pink, non‐tender soft tissue mass/(4.3 × 3.2) cm.	Maxillary alveolar ridge.	Electric cauterization.	4 months.	Healing uneventful, no recurrence.
15.	Patil RN et al (2017)^30^	Newborn/ Female.	Pedunculated Soft tissue Mass/ (2 × 2) cm.	Maxillary alveolar ridge.	Excision.	Follow‐up time is not mentioned.	Not known.
16.	Rech BO et al. (2017)^31^	Newborn/Female.	Solitary, firm, pedunculated nodular mass/Diameter–3 cm.	Maxillary anterior alveolar ridge.	Excision.	9 years.	Healing uneventful, no recurrence.
17.	S Shojaei et al (2018)^32^	Newborn/Female.	Pedunculated soft tissue mass/(10 × 8 × 4) mm.	Mandibular anterior alveolar ridge.	Excision.	Follow‐up time is not mentioned.	Not known.
18.	P Gardener et al (2018)^33^	Newborn/Female.	Pedunculated Soft tissue mass/(1.5) cm.	Anterior mandibular alveolus.	Excision.	3 weeks.	Healing uneventful/No recurrence.
19.	KS Rodrigues et al (2019)^34^	Newborn/Female	Nodular exophytic lesion/size not mentioned.	Maxillary anterior alveolar ridge.	Excision.	Follow‐up time is not mentioned.	Not known.
20.	BO Castano et al (2020)^35^	3‐week‐old/Female.	Swelling, pedunculated mass/(2 × 2) cm.	Maxillary right anterior dentoalveolar segment.	Excision.	1 month.	Healing uneventful/No recurrence.
21.	R Atheetha et al (2021)^36^	18‐day‐old/Female.	Soft tissue overgrowth/(1 × 1) cm.	Maxillary anterior gingiva.	Excision.	Follow‐up time is not mentioned.	Not known.
22.	Gan J et al. (2021)^37^	2‐day‐old/Female.	Multiple pedunculated soft tissue lesions/(Size of largest–3 cm in diameter).	1 on maxillary and 1 on mandibular alveolar ridge).	Mandibular‐ Excision Maxillary‐ observation (as the lesion was only 0.5 cm in diameter).	6 months.	Healing uneventful, no recurrence. Maxillary lesion‐ Spontaneous regression, no recurrence.
23.	Rattan A et al. (2021)^38^	Newborn/Male.	Solitary, non‐tender, firm, smooth, sessile mass/(3.5 × 2.6) cm.	Mandibular alveolar ridge.	Excision.	Follow‐up time is not mentioned.	Healing uneventful, recurrence data not mentioned.
24.	Present case	Newborn/Female.	Solitary, pedunculated mass/(2.3 × 1.8 × 1.4) cm.	Mandibular alveolar ridge.	Excision.	The patient did not report for follow‐up,	Healing was uneventful.

A wide range of immunohistochemical (IHC) markers could be used in CGCT including S100, CD 68, CD 105, podoplanin, and vascular endothelial growth factor (VEGF). But all cases of CGCT do not show positivity for all these IHC markers speaks for its enigmatic tissue of origin.^5^, ^11^ S100 has been positive in cases of adult granular cell tumor, rather than CGCT, which suggests that CGCT may have a different tissue of origin and the absence of Schwann cells.^11^, ^12^

The differential diagnosis for this particular entity includes granular cell tumor, congenital hemangioma, melanotic neuroectodermal tumor of infancy (MNTI), embryonal rhabdomyosarcoma, infantile myofibroma, and peripheral odontogenic fibroma.^4^, ^13^, ^14^, ^15^ The clinicopathologic and immunohistochemical attributes of CGCT and its differential diagnosis are mentioned in Table 2.

**TABLE 2:** Clinicopathologic attributes of congenital granular cell tumor (CGCT) with its differential diagnosis^4^, ^13^, ^14^, ^15^

Lesion	Clinical features	Histopathologic features	Immunohistochemistry
Congenital granular cell tumor	**Sex–**F > M, **Age–**newborn **Predilection site–**Gingiva/Alveolar ridge of anterior maxilla. Presentation‐ Pedunculated/sessile mass.	Sheets of round, oval, polyhedral cells with basophilic nuclei and granular eosinophilic cytoplasm.	**Positive–**vimentin **Negative–**S 100
Granular cell tumor	**Sex–**F > M, **Age–**30–60 years **Predilection site–**Tongue. **Presentation–**Solitary nodule on the anterior tongue.	Sheets of granular, eosinophilic cells with pseudoepitheliomatous hyperplasia of the overlying squamous epithelium.	**Positive–**S 100, CD 68
Congenital Hemangioma	**Sex–**M = F, **Age–**Newborn **Predilection site–**Scalp, face. **Presentation–**Solitary/Multiple soft tissue mass, pulsatile.	Multiple plump endothelial lined blood vessels in a sparsely cellular stroma. Presence of mast cells noted.	**Positive–**CD 34, VEGF
Melanotic neuroectodermal tumor of Infancy (MNTI)	**Sex–**M > F, **Age**–Infant **Predilection site–**Maxilla. **Presentation–**Painless, expansile, pigmented mass.	Composed of alveolar spaces lined by cuboidal or polygonal cells containing pale, eosinophilic cytoplasm, it also has melanin pigment.	**Positive–**Cytokeratin, NSE, HMB 45, Synaptophysin
Infantile Myofibroma	**Sex–**M > F, **Age–**Infant–6 years **Predilection site–**Buccal mucosa and Tongue. **Presentation–**Soft tissue mass.	Interlacing fascicles of spindle‐shaped cells resembling fibroblasts or smooth muscle.	**Positive–**Vimentin, SMA
Embryonal rhabdomyosarcoma	**Sex–**M = F, **Age–**2–6 years **Predilection site (intraoral)–**Tongue, buccal mucosa and palate. **Presentation–**Extensive swelling, smooth in consistency.	It is a malignant tumor of striated muscles, containing a multiphasic population of cells, pleomorphism also noted among the tumor population.	**Positive–**Desmin, Myogenin
Peripheral odontogenic fibroma	**Sex–**F > M, **Age–**Variable **Predilection site**–Mandible. **Presentation–**Exophytic, gingival swelling.	Cellular connective tissue with strands of odontogenic epithelium.	**Positive–**Cytokeratin 14,19 (in the region of odontogenic epithelium only)

Abbreviations: F, Female; M, Male; NSE, Neuron‐specific enolase; SMA, Smooth muscle actin.

## 4 CONCLUSION

Congenital granular cell tumor is a distinctive entity rarely encountered mostly in infants. The exact tissue of origin, course, and progression of this entity is obscure, which invokes further research. The take‐home message from this case report is prenatal diagnosis is important for treatment planning and depending on the size and location of the lesion to prevent any further complications. Although recurrence is rare, and malignant transformation has not been reported, periodic follow‐up of the patient should be performed.

## CONFLICT OF INTEREST

None.

## AUTHOR CONTRIBUTIONS

Snehashish Ghosh drafted the manuscript, revised, and edited it. Arpita Singh has done the literature review, edited the figures. Anuja Panthee has revised the manuscript. Anjani Kumar Yadav has assisted in literature review and editing of the manuscript. All the authors have reviewed the paper and approved the final version of the manuscript.

## ETHICAL APPROVAL

Ethics approval was not required from the institution, in accordance with our country's law, as this was a case report.

## CONSENT

Written informed consent was obtained from the father of the patient (as the patient was a neonate) to publish this report in accordance with the journal's patient consent policy.

## Data Availability

The data that support the findings of this article are available from the corresponding author upon reasonable request.
